# Typhoid Fever

**Published:** 1894-10

**Authors:** W. N. Rogers

**Affiliations:** Belton, Texas


### Correspondence.

### TYPHOID FEVER.

BELTON, TEXAS, August 30, 1894.

*Editor Texas Medical Journal:*

During the past eight weeks we have had what might be termed a mild epidemic of continued fever in this section. I have treated during that time about sixteen cases, several of which were well defined typhoid fever. The majority, however, were of milder form and presented less of the symptoms of typhoid. I am rather inclined to the opinion that they are all typhoid of a mild or modified type, but of this I cannot be positive.

The duration of the disease ranged from twenty-one to thirty-five days, except one case, which terminated fatally on the eighth day. This was a boy eight years old, of feeble constitution, with severe diarrhœa and high fever from the fourth day. The nervous system was severely taxed, and the heart's action overwhelmed from the poison of the disease. It is of the treatment I wish to speak more than anything else. You and the readers of the JOURNAL are probably aware of the fact that I am an enthusiastic supporter of the intestinal antiseptic treatment of typhoid fever, and that I have been for the past ten years. I am more convinced of the superiority of this treatment the longer I observe it; and I am fully sustained in this by our best authorities on this subject. In the 1893 Annual of the Universal Medical Sciences we are advised to make it a routine treatment; of course this does not preclude other lines of treatment.

I have in all of my cases kept the bowels in an aseptic condition as nearly as is possible by the use of bismuth, salol, salicylate soda, creosote, tonic doses of calomel at intervals of two or three days, and naphthalin. By this line of treatment, I prevent to a very great extent all of those bad symptoms and complications which endanger the life and comfort of the patient. I have never had a serious hemorrhage in my practice since I have been using this plan of treatment, and only two slight hemorrhages at all in ten years, while there has been a great number of fatal and severe cases where this has been neglected.

I never have seen any of the severe complications, such as abscesses, thrombi, neuritis, otitis, periostitis, and others too numerous to mention, where the bowels have been kept in an aseptic condition.

It is a well known fact that these complications are produced by the poison of the disease being absorbed into the system, and to prevent this poisoning, we must prevent the formation of them in the intestinal tract. This can practically be done by the antiseptics above mentioned being thoroughly administered from the beginning of the treatment.

Besides the appetite and digestion are kept in the very best possible condition under this plan. Tympanitis, that very distressing symptom, is controlled in this way by preventing that fermentation from which it is produced.

It is surprising the number of physicians who are yet treating typhoid fever in the same way it was treated thirty or forty years ago, when the literature of the day is strongly in favor of the modern scientific method of treatment, and while the results of the ancient method are so unsatisfactory, with all the long train of bad symptoms and complications.

W. N. ROGERS, M. D.

